# Insights into the pathogenesis of vein graft disease: lessons from intravascular ultrasound

**DOI:** 10.1186/1476-7120-2-8

**Published:** 2004-07-21

**Authors:** Gavin J Murphy, Gianni D Angelini

**Affiliations:** 1 Bristol Heart Institute, University of Bristol, Bristol, BS2 8HW, UK

**Keywords:** Coronary artery bypass grafting, intravascular ultrasound, saphenous vein graft

## Abstract

The success of coronary artery bypass grafting (CABG) is limited by poor long-term graft patency. Saphenous vein is used in the vast majority of CABG operations, although 15% are occluded at one year with as many as 50% occluded at 10 years due to progressive graft atherosclerosis. Intravascular ultrasound (IVUS) has greatly increased our understanding of this process. IVUS studies have shown that early wall thickening and adaptive remodeling of vein grafts occurs within the first few weeks post implantation, with these changes stabilising in angiographically normal vein grafts after six months. Early changes predispose to later atherosclerosis with occlusive plaque detectable in vein grafts within the first year. Both expansive and constrictive remodelling is present in diseased vein grafts, where the latter contributes significantly to occlusive disease. These findings correlate closely with experimental and clinicopathological studies and help define the windows for prevention, intervention or plaque stabilisation strategies. IVUS is also the natural tool for evaluating the effectiveness of pharmacological and other treatments that may prevent or slow the progression of vein graft disease in clinical trials.

## Introduction

The success of coronary artery bypass grafting (CABG), although the gold standard for the treatment of multivessel coronary artery disease, is limited by poor long-term vein graft patency [1]. Early vein graft thrombosis (within 1 month) occurs in up to 15% of vein grafts due to graft spasm or technical error [2,3], whilst late vein graft failure occurs as a consequence of early neointimal hyperplasia with later superimposed atheroma, so called 'vein graft disease' [1,4] and as many as 50% of all vein grafts are occluded at 10 years post surgery [5,6]. Despite the superiority of arterial graft patency over that of vein grafts, the multivessel nature of coronary artery disease and ready availability of saphenous vein still result in its use in over 70% of CABG procedures [7]. The alternative treatment modality for multivessel coronary atheroma, percutaneous coronary artery angioplasty and stenting (PCI), has traditionally suffered from even worse long-term results compared to CABG, due to high early restenosis rates; over 30% within 1 year [8]. This results in more frequent and more rapid return of symptoms and major adverse cardiac events (MACE) with PCI compared to CABG, necessitating more repeat revascularisation procedures [8]. The apparent success of new drug eluting stents has challenged this paradigm however. Rapamycin (a macrolide antibiotic) and the taxane, paclitaxel, two agents with potent antiproliferative properties, eluted from intracoronary stents, have dramatically reduced restenosis rates, MACE and reintervention rates in clinical trials [9,10], to the point where the superiority of CABG is now being seriously challenged [11]. This represents the clinical application of intensive research into the mechanisms of atherosclerosis and restenosis and strategies for their prevention over the last decade. Conversely, CABG has suffered from its apparent success, and with some exceptions [12], there have been comparatively few attempts to prevent or inhibit the progression of vein graft disease in CABG patients, a condition that must change. Vein graft disease differs from arterial atherosclerosis in that its natural history is much shorter and the date of onset is clearly defined, i.e. graft implantation. This process is therefore potentially amenable to strategies that may inhibit its progression.

Although the cellular and molecular mechanisms underlying vein graft disease have been systematically investigated, the time course and development of this process in patients after coronary bypass has only recently been defined as a consequence of the increasing use of intravascular ultrasound (IVUS). Quantitative coronary angiography, traditionally the predominant imaging modality used to assess the severity of vein graft disease underestimates the severity of vein graft remodeling and athermanous plaque development by measuring the vessel lumen in only two dimensions [13,14]. In contrast, the tomographic IVUS image enables visualization of the full circumference of the vessel wall [15] allowing measurement of wall thickening, vein remodeling and atherosclerotic plaque size, distribution, and composition [15]. This results in the detection of diffuse atherosclerotic plaque, compensatory vessel enlargement and preservation of the luminal diameter even in angiographically normal vessels [13,14,16]. IVUS findings in vein grafts also show good correlation with histological findings in clinicopathological studies [17]. The purpose of this review is to summarize our current understanding of the natural history of vein graft disease from IVUS studies, correlate this with the findings of experimental and clinicopathological studies, and, finally to consider how this knowledge, may be used to target prevention or treatment strategies.

### Early changes in saphenous vein bypass grafts; wall thickening and adaptive remodelling

**Figure 1:**
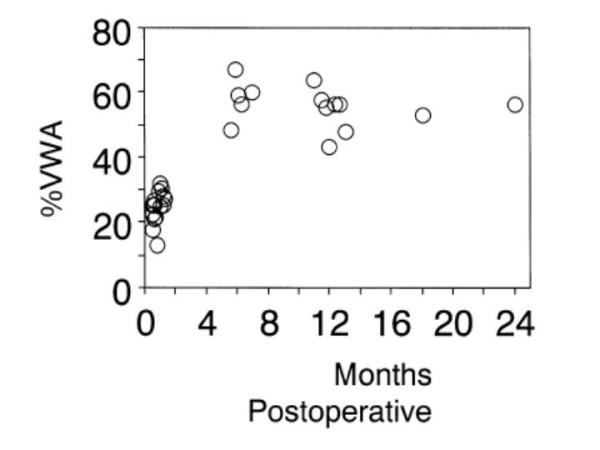
Increases in wall thickness versus time after surgery. Wall area expressed as a percentage of total vessel area (%VWA) exceeded 40% and reached a plateau state after 6 months in angiographically normal vessels. Reproduced with permission from Higuchi et al, Heart Vessels 2002, 17:57–60, Springer – Verlag, Heidelberg, Germany [16].

Glagovian remodeling was first described as a radial enlargement of the entire cross sectional area of a vessel in response to intramural atheroma [18]. First identified in humans in post-mortem studies it was only with the widespread use of IVUS that its central role in atherosclerosis, post angioplasty restenosis, transplant vasculopathy and vein graft disease was realised [15]. Currently, the term not only applies to vessel enlargement, but also shrinkage, where in the presence of underlying plaque it becomes an important determinant of lumen loss [19]. In vein grafts, early after implantation, increases in overall vessel cross sectional area preserve luminal size despite significant increases in wall thickness [16]. IVUS measured parameters of vessel remodeling and wall thickening in vein grafts pre, or early post implantation versus later periods are described in Table 1. Vein graft dimensions within one month of implantation are remarkably similar to those in grafts prior to implantation [16,20,24], with significant wall thickening having occurred by six months, even in angiographically normal grafts [14,16]. Other studies have demonstrated wall thickening as early as 3 weeks to 3 months after CABG [23,24]. These changes are diffuse and concentric, and are observed from the aortic root to the coronary anastomosis [23,24]. Higuchi and colleagues compared IVUS measurements of 15 vein grafts performed within 1 month postoperatively with 14 vein grafts after 6 months postoperatively. This showed that significant wall thickening had occurred by 6-months, accompanied by compensatory enlargement, and preservation of the graft luminal diameter (Table 1), however wall thickening appeared to reach a plateau after 6 months with preservation of lumen area (Figure 1), suggesting that early remodeling responses may stabilize in the absence of atherosclerotic development [16].

**Table 1:** Early adaptive changes and neointima formation in saphenous vein grafts

**Study Reference**	**Grafts / Patients**	**Pre implantation to 1 month (mm^2^)**				**>12 months (mm^2^)**						
		Lumen	Wall Area	Vessel CSA	%wall area	Lumen	Plaque area	Wall Area	EEL area	Vessel CSA	% plaque area	%wall area
**Nishioka et al 1996 [20]**	43/42	16.5 ± 5.7	7.4 ± 2.1	23.9 ± 7.3	32.3 ± 7	8.9 ± 2.7	10.0 ± 5.3	15.2 ± 5.8	18.8 ± 7.5	24.0 ± 7.8	51 ± 10	63 ± 7
Ge et al 1999** [21]	43/43								12.6 ± 4.0 – 19.0 ± 9.7		64.5 ± 15.5	
Hong et al 1999** [22]	104/93					12.0 ± 4.2 – 3.8 ± 1.9	7.2 ± 4.1 – 13.9 ± 4.9	10.0 ± 3.0 – 20.3 ± 6.5	16.7 ± .9 17.8 ± 6.1	20.8 ± 5.1 – 24.1 ± 7.8	30 ± 5 – 79 ± 9	45 ± 5 – 83 ± 7
**Higuchi et al 2002τ [16]**	47	16.2 ± 5.5	5.3 ± 2.0	21.6 ± 7.1	24.9 ± 5.0	12.8 ± 4.6		15.8 ± 5.2		28.8 ± 8.8		55.7 ± 6.8

**values represent range from reference segment to focal stenosis, τ angiographically normal vein. Vessel CSA, (cross sectional area) measured by tracing the outer border of the whole vein graft, *Wall area*, *Vessel CSA *minus lumen area. *Percent wall area *was calculated as the wall area divided by Vessel CSA. In situ veins do not have an external elastic membrane however; arterialized saphenous vein grafts develop a sonolucent zone, which has been reported to represent media. The EEL (external elastic membrane) area is measured by tracing the outer border of this sonolucent zone. Plaque area is calculated as external elastic membrane minus lumen area. Percent plaque area is calculated as plaque area divided by external elastic membrane area; this has also been called the plaque burden. Plaque burden and percent wall are closely correlated.

IVUS changes correlate closely with the findings of experimental and clinical studies. In porcine saphenous vein bypass grafts, in the first week after grafting, adventitial medial and neointimal thickening occurs as a consequence of increased shear stress, surgical preparative injury and the subsequent activation of multiple growth factor and cytokine cascades. This is associated with the infiltration of inflammatory cells, medial smooth muscle cell proliferation and migration to form a neointima [25]. Adventitial myofibroblast proliferation and extracellular matrix deposition also results in the formation of a thick neoadventitia [26]. These myofibroblasts migrate through all the layers of the vessel wall, where subsequent extracellular matrix deposition contributes to overall wall thickening [27]. A similar distribution of cytoskeletal proteins characteristic of myofibroblasts is observed in explanted human saphenous vein grafts suggesting that similar mechanisms occur in man [27]. After the first week, wall thickening in porcine vein grafts occurs largely due to extracellular matrix deposition (fibrosis) and neointimal smooth muscle cell proliferation, however this thickening plateaus after one month [26].

The early changes seen in the vessel wall of vein grafts are similar to those seen during vessel remodeling in atherosclerotic coronary artery segments [19]. In normal arteries, remodeling is a homeostatic response to changes in flow and circumferential stretch, with compensatory enlargement and wall thickening normalizing shear stress and wall tension in response to higher blood pressures and flow velocities respectively. Outward remodeling in response to increased flow is largely dependent on shear-responsive endothelial production of nitric oxide and the gelatinase matrix metalloproteinases (MMPs) MMP-2 and MMP-9 [28,29]. MMPs are central to the turnover of the extracellular matrix, altering cell-cell interactions, modifying the extracellular milieu and permitting the movement and division of cells. Increased MMP production, with extracellular matrix degradation is a feature of the infiltration of inflammatory cells as well as the migration of smooth muscle cells and myofibroblasts [30,31], and this may also contribute to the remodeling process [19].

### Late changes in vein grafts: atherosclerosis and pathological remodelling

Early vein graft changes can be viewed as adaptive, however they also predispose the graft to later accelerated graft atherosclerosis [32]. Several components of the extracellular matrix that are abundant in diffuse fibrous intimal hyperplasia may increase the residence of atherogenic molecules, and promote the development of lipid-laden lesions [33,34]. Similarly, myofibroblasts are associated with contractile responses as part of wound healing [35] and it has been hypothesized that dissemination of these cells throughout all layers of the vein graft may be central to later inadequate or constrictive vessel remodeling [36,37].

Risk factors for, and the microscopic appearance of vein graft atherosclerosis are largely similar to those in coronary arteries and it is reasonable to suggest that similar pathological mechanisms are at work, however these occur over a much more rapid time course in vein grafts [1,4]. Atheromatous plaque is detected by IVUS as early as eight to ten months post grafting [38] in association with both expansive and constrictive remodelling [22] (Table 2). This is much earlier than originally suggested by angiography [5,6]. Early IVUS studies disagreed as to the nature of vein graft remodeling, with some studies reporting expansive remodeling [21,38] whilst others did not [20]. This confusion was most likely due to the small sample sizes in these early studies however (Table 2). Hong et al [22] used IVUS to assess the extent and direction of remodeling in 104 grafts in 93 patients, the largest analysis of diseased vein grafts published to date. In individual lesions, they defined remodeling by comparing the area within the EEL at the site of stenosis to that of a reference point. Positive remodeling was defined as a stenosis/ reference EEL area ratio >1.1, intermediate remodeling as a ratio 0.9 to 1.1, and negative remodeling as a ratio <0.9 [39]. All three processes were shown to occur, sometimes even within the same vessel. Overall plaque burden was greater in positively remodeled segments compared with intermediate or negative remodeling, whilst lumen area was preserved in all groups [22]. This is similar to changes that occur in atherosclerotic coronary arteries [40]. Mendellson and colleagues identified expansive remodeling in 98.5% of 24 vein graft lesions studied (Table 2). They showed that lumen area did not change with increasing percent area stenosis for vessels with ~30% of their area occupied by plaque (p = NS), however, for segments with >30% of the vessel area occupied by plaque, there was an inverse relation with the lumen area [38]. In this group (with >30% effective plaque area stenosis), the lumen area decreased as the percentage of vessel area occupied by plaque increased. Again these were the same changes as those noted in coronary arteries [41]. They suggest that "compensatory" enlargement mechanisms are very effective for early atherosclerotic lesions; but as lesions become more progressive, these mechanisms can no longer compensate, and lumen narrowing occurs. This does not explain constrictive or negative remodeling however and the mechanisms underlying this process remain unclear. In addition to the role of myofibroblasts, the formation of a dense neoadventitia with extensive collagen deposition has been implicated as a mechanical barrier to vessel enlargement, with later remodeling of this fibrous tissue ultimately resulting in vessel shrinkage [37]. In atherosclerotic arteries negative or inadequate remodeling is more common in insulin-using than non-insulin-using diabetics [42], more common in smokers compared with nonsmokers, and less frequent in patients with hypercholesterolaemia [43]. Mendellson et al showed that expansive vein graft remodeling was independent of graft age, insertion site, plaque eccentricity, patient age, or gender [38]. Altered local haemodynamics can also affect remodeling. Low shear predisposes the inner curves of tortuous segments to develop atheroma and may impair outward remodeling in a similar manner [44,45]. Alternatively, medial thinning as a consequence of atherosclerotic plaque development may result in bulging of the vessel wall due to diminished structural support at the site of the plaque [46]. Plaque volume often correlates with the level of inflammatory infiltrate, which again may contribute to expansion by promoting collagen lysis and deposition of loose myxoid extracellular matrix [47]. This is thought to underlie the propensity for such plaques to rupture and explain the association between expansive remodeling and unstable coronary symptoms [19].

**Table 2:** Late remodeling in atherosclerotic vein grafts

	**Grafts /** **Patients**	**Latency** **(years)**	**Lumen area**		**Intimal and medial(plaque)** **area**		**External elastic lamina area**	
			Reference	Stenosis	Reference	Stenosis	Reference	Stenosis
**Mendellson et al 1995 [38]**	21/19	0.8–16	14.6 ± 7.5	7.1 ± 4.5	5.6 ± 3.4	18.3 ± 7.0	20.2 ± 8.5	25.4 ± 8.2
**Nishioha et al 1996 [20]**	43/42	3–12	15.7 ± 6.8	5.0 ± 1.5	3.2 ± 1.5	13.7 ± 6.3	18.9 ± 7.0	18.7 ± 7.3
**Ge et al 1997 [21]**	43/43	1–6					12.6 ± 4.0	19.0 ± 9.7
**Hong et al 1999 [22]**								
**Negative remodeling**	104/93	1.2–20.7	12.5 ± 4.0	3.6 ± 2.0	5.0 ± 2.4 -	10.9 ± 6.2	17.8 ± 7.9	14.2 ± 8.1
**Positive remodeling**			11.8 ± 3.5	4.0 ± 1.8	5.1 ± 1.4	15.3 ± 4.7	16.7 ± 4.6	19.4 ± 6.2

Remodeling can be defined as the ratio of the EEL at the site of the stenosis to that of the reference point. Positive remodeling is defined as a stenosis/ reference EEL area ratio >1.1, intermediate remodeling is defined as a ratio 0.9 to 1.1, and negative remodeling as a ratio <0.9.

### The future

IVUS studies have clearly shown that early 'adaptive' or pathological changes occur within weeks of grafting and that occlusive atheroma, in susceptible individuals occurs within 1 year. IVUS studies have therefore defined the window in which strategies to inhibit vein graft disease might be effective. Furthermore, in addition to its advantages over coronary angiography, IVUS in vein grafts has been shown to be both accurate [12,16] and reproducible [20,22] making it the obvious investigational tool to explore the effectiveness of these strategies on in clinical trials. IVUS measurements correlate closely with clinicopathological findings [12] and can detect standardised differences of >1 in studies of saphenous vein grafts versus ungrafted vein as well as in vein grafts early post implantation (within 1 month) compared with later periods [16,20]. In addition, Nishioka and colleagues [20] demonstrated that for the measurement of lumen area, the mean difference between two observations by the same observer was 3.1%, with a range of 0% to 9.0%. Between two observers, the mean difference was 3.8%, with a range of 1.0% to 5.6%. This reproducibility facilitates not only accurate comparisons between groups of patients but also assessment of the effects of intervention on grafts in longitudinal studies.

The ability to manipulate vein grafts *ex vivo *prior to implantation using pharmacological or other methods that may inhibit subsequent disease is a feature unique to vein graft disease. There are many examples of this being successfully achieved in experimental models. Pre-treatment with rapamycin [48], paclitaxel (GD Angelini, unpublished observations) and the intracellular calcium dependant ATPase inhibitor, thapsigargin [4], have been shown to significantly inhibit the progression of vein graft disease in experimental models in vivo. Oral agents, such as NO donating aspirins [49] and endothelin antagonists have also been shown to be effective in porcine vein grafts in vivo [50] as has the application of a porous external polyester stent which inhibits neointima formation and promotes expansive remodelling in the absence of vein wall thickening [51]. Targeted gene transfer is another attractive option, with viral transfer of E2F-decoy oligonucleotides inhibiting vein graft failure after peripheral arterial reconstruction in clinical trials [52]. Stabilisation of vein grafts that have undergone early adaptive changes, but have yet to develop a large plaque burden is also a possibility. Risk factor modification such as cessation of smoking and aggressive lipid lowering [62–64] has been shown to improve long-term graft patency on angiography. The effect of other interventions such as antiplatelet therapy on the progression of vein graft disease have not been evaluated in IVUS studies however. Identification of patients with a large plaque burden on IVUS, but otherwise angiographically normal vein grafts may also enable targeted plaque stabilisation therapy.

## Conclusion

IVUS has significantly contributed to our understanding of vein graft failure. It also serves as the natural tool for the development of clinical strategies that may lead to significant improvements in vein graft patency and more importantly for better long-term quality of life and longevity for patients with coronary artery disease. The introduction of clinical trials to address this Achille's heel of coronary bypass surgery are long overdue.

## Competing interests

None declared.

## Authors contributions

G Murphy performed the literature review and prepared the manuscript. G Angelini conceived the idea for the manuscript and prepared the manuscript.
